# Effects of Ultratrail Running on Neuromuscular Function, Muscle Damage and Hydration Status. Differences According to Training Level

**DOI:** 10.3390/ijerph18105119

**Published:** 2021-05-12

**Authors:** Francisco Pradas, David Falcón, Carlos Peñarrubia-Lozano, Víctor Toro-Román, Luis Carrasco, Carlos Castellar

**Affiliations:** 1 ENFYRED Research Group, Faculty of Health and Sports Sciences, University of Zaragoza, 22001 Huesca, Spain; franprad@unizar.es (F.P.); dfalcon@unizar.es (D.F.); carlospl@unizar.es (C.P.-L.); castella@unizar.es (C.C.); 2 Department of Corporal Expression, Faculty of Health and Sports Sciences, University of Zaragoza, 22001 Huesca, Spain; 3 School of Sport Sciences, University of Extremadura, Avenida de la Universidad s/n, 10003 Cáceres, Spain; 4 BIOFANEX Research Group, Department of Physical Education and Sport, Faculty of Education Sciences, University of Seville, 41004 Seville, Spain; lcarrasco@us.es

**Keywords:** trail running, jump, neuromuscular, hydration

## Abstract

The status of trail running races has exponentially grown in recent years. The present study aimed to: (a) evaluate the acute response of ultratrail racing in terms of neuromuscular function, muscle damage and hydration status; (b) analyze if responses could differ according to training levels. Twenty runners participated in the present study. The participants were divided into amateur training level (*n* = 10; 43.30 ± 4.52 years) or high level competitors (*n* = 10; 41.40 ± 6.18). Neuromuscular response (squat jump, countermovement jump and Abalakov jump), muscle damage (alanine aminotransferase, bilirubin, creatine kinase and leukocytes) and hydration status (sodium and creatinine) were evaluated before and after the Guara Somontano Ultratrail Race (108 km distance, with an accumulated slope of 5800 m). The height and power achieved by vertical jumps were lower after the race (*p* < 0.001). The post-race muscle damage and creatinine parameters increased in both groups (*p* < 0.001). The high-level group obtained lower percentages of change in squat jump and countermovement jump than the amateur-level group (*p* < 0.05). However, the increase in creatinine was greater for the high-level group (*p* < 0.05). Ultratrail racing reduces neuromuscular function and increases muscle damage. High-level runners showed less neuromuscular fatigue compared to amateur ones.

## 1. Introduction

Ultratrail is a form of very long-distance mountain racing included in trail running. Given its extreme characteristics, this kind of racing can be considered very taxing, or even dangerous, for our organism [1]. Ultratrail racing is performed on routes with adverse orography. Its distance exceeds that of marathons [2,3], and ranges between 80 km and 300 km [4]. The fact that ultratrail runners face unstable environmental and weather conditions for long periods of time, and constant changes in the topography of land and altitude [4], with considerable positive and negative slopes [5], is also emphasized.

Despite trail running races being extremely hard, the organization of the sport has exponentially grown worldwide in recent times [6,7,8], and Spain is no exception [9]. The marked demand expected today of such races has drawn more participants, to the extent that the number of runners in races must be limited, which has led to some runners becoming professionals with enhanced ultratrail race performance [10]. 

Ultratrail races are multifactorial events that include physiological, neuromuscular, biomechanical and psychological variables [2]. Depending on exertion while running these races, research works indicate that according to ventilator thresholds (VT), in physiological terms these races place mean training intensities at 85.7% for zone I (<VT1), 13.9% for zone II (VT1–VT2) and 0.4% for zone III (>VT2) [11]. Cardiac responses during races lasting between 14 and 7 h present intensities of 64% [12] and 82% [13] of maximum heart rate (HR_max_), respectively. Different research works have analyzed lactate kinetics during such races, and have obtained results that are below the onset of blood lactate accumulation (OBLA) [14,15]. Finally, as regards the psychophysiological component, values between 13 and 14 points have been obtained on the rate perceived exertion (RPE) scale [16]. 

In ultratrail endurance races, optimum performance might depend on high maximal oxygen consumption (VO_2max_) levels, high VO_2max_ fraction utilization percentages and low oxygen transport cost [2]. Along these lines, it has been demonstrated that maximum aerobic power can be a limiting factor to running on positive slopes [17], which are usual for ultratrail routes. When considering the types of routes that ultratrail races are organized along, it would appear that adapting to exertions, in which eccentric-type activations predominate, could be important for facing the ascents and constant loads that come into play when running on irregular sloping surfaces. The eccentric capacity to maintain maximum racing speed would be a determining factor in zones with negative slopes [18].

The increasing numbers of both participants and ultratrail races have favored different research studies being performed in an attempt to analyze the impact that this racing has on health. Indeed, ultratrail races have been associated with a negative energy balance [13], muscle harm and inflammation [19,20], neuromuscular fatigue [21,22], cardiac dysfunctions, myocardial damage [13] and dehydration [1].

The relationship between hydration status and performance has been described [23,24]. Hydration status is directly related to physical performance [25]. Previous studies have reported the consequences of dehydration at the physical level, which can negatively affect sports performance [26,27]. A small decrement in hydration status impaired physiologic function and performance during a trail running race [28]. In addition, dehydration status could negatively affect the neuromuscular response [29].

Bearing in mind the marked physical stress that the organism is submitted to while running an ultratrail race, it can be stated that running such races in an unplanned and uncontrolled manner can have very negative consequences for the health of the people practicing this sport [1]. Indeed, knowing the limits of the organism’s physiological responses while practicing long intense exercise is necessary to avoid resorting to urgent medical assistance [30]. However, the impact of an ultratrail race could differ from one individual to another because they have distinct training levels. This means that more studies need to be conducted to elucidate this statement [31,32]. The impacts of such races could be very different depending on the participants’ training levels, owing to their distinct physiological profiles [33]. Hence, the objectives of the present study were to: (a) evaluate the acute responses to an ultratrail race in terms of neuromuscular function, muscle damage and hydration status; (b) analyze if responses could differ according to training levels.

## 2. Materials and Methods

### 2.1. Participants

Twenty runners participated in this quasiexperimental study, and were divided into an amateur-level group (*n* = 10) and a high-level group (*n* = 10) according to their training levels [34]. The high-level group was made up of runners who trained for a minimum of 8 h/week and achieved positive accumulated slopes of more than 50,000 m in the 12 months before the test. The amateur group was formed by those runners who did not meet any of the set requirements. The study was conducted on a population of 176 male participants who completed the race. The minimum sample size was 20 participants (confidence level = 95%; margin of error = 21%). The characteristics of the participants in each group are found in Table 1. 

All the participants were informed about the study purpose and signed a consent form before enrolling. The protocol was reviewed and approved by the Ethical Committee of Clinical Research of Aragon (Spain) following the guidelines of the Helsinki Declaration of Ethics, updated at the World Medical Assembly in Fortaleza (2013) for research involving human subjects. A code was assigned to each participant to collect and treat samples in order to maintain their anonymity.

In order to participate in this study, the participants had to meet the following inclusion criteria: (a) male; (b) finished the race route; (c) had no injuries after a race in at least the 2 months prior to the test; (d) not on a special diet or any pharmacological treatment; (e) no cardiovascular or metabolic diseases.

### 2.2. Procedures

**Figure 1:**
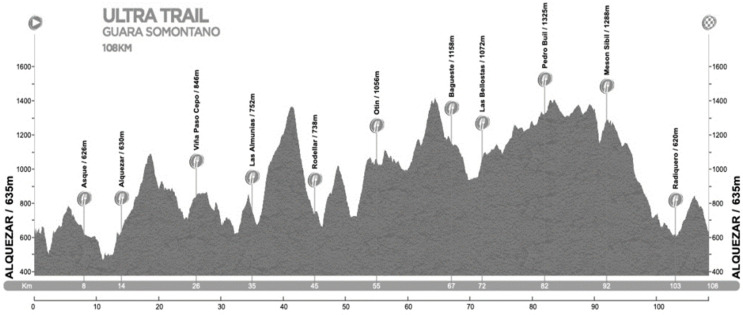
Race profile (taken from the organization’s website).

This study was carried out while organizing the Guara Somontano UltraTrail Race (GSUR) (Figure 1), which took place in the town of Alquézar (Huesca, Spain). The total race distance was 108 km, with 5800 meters of positive accumulated slope. The maximum permitted time to finish it in was 24 h. During this race the mean temperature was 14 ± 4.4 ℃ and relative humidity was 57 ± 16.1% (Figure 1).

The week before competition, the participants underwent body composition and physical performance evaluations. In parallel to these evaluations, the runners completed an open *ad hoc* survey with supplementary information about their experience, and the characteristics of their training over the past year (h/week and accumulated slope). To determine hydration during races, the volumes of liquids drunk by racers were calculated at both supply points and throughout the race according to individual drinking frequency. Volunteers recorded the amounts of water ingested at each supply point using two 250 cc bottles as a reference. At each refreshment point, two 250 cc bottles were given to each participant.

### 2.3. Anthropometric Measurements

The participants’ morphological characteristics were evaluated in the morning and always under the same conditions. Body height was measured to the nearest 0.1 cm using a wall-mounted stadiometer (Seca 220, Seca, Hamburg, Germany). Body weight was measured to the nearest 0.01 kg on calibrated electronic digital scales (Seca 769, Seca, Hamburg, Germany), barefooted. A Holtain© 610ND (Holtain, Crymych, Wales, UK) skin fold compass, accurate to ±0.2 mm and a tape (Seca 212, Seca, Hamburg, Germany) with an accuracy of ±1 mm were employed to take anthropometric assessments. The obtained anthropometric measurements were height, weight, six skin folds (abdominal, suprailiac, subscapular, tricipital, thigh and leg) and perimeters (arm and leg in a relaxed 90° position). The equations of Yushaz were used to calculate the percentage of fat [35] and the equation according to Lee to determine the percentage of muscle [36]. All the measurements were taken by the same operator, who was skilled in kinanthropometric techniques, and in accordance with the International Society for the Advancement of Kinanthropometry recommendations [37]. Body weight was recorded 2 h before and immediately after the race finished.

### 2.4. Physical Performance Evaluation

In order to determine the corresponding physical performance values, a progressive and maximum laboratory test was done on a treadmill (Pulsar, h/p/cosmos®, Nussdorf, Germany). The test was run on a 1% slope and began at a speed of 8 km/h, which increased 1 km/h every minute. Before the test began, the participants warmed up for 5 min on the treadmill operating at a speed of 6 km.h^−1^. Respired gases were collected with an Oxycon Pro analyzer (Erich Jaeger GmbH, Hoechberg, Germany). A pulsometer (Vantage M, Polar, Finland) was used to evaluate the maximal heart rate.

### 2.5. Neuromuscular Function

Neuromuscular function was evaluated by different jumping tests: squat jump (SJ), countermovement jump (CMJ) and Abalakov jump (ABK) [38,39]. The former tests were chosen to measure the neuromuscular function of leg extensor muscles, because they can perform jumps with a high degree of reliability [40,41]. A jump mat system (Chronojump Boscosystems, Barcelona, Spain) was employed to measure the height and time during jumps. Three attempts were made for all jump types; there was a 30-s rest between jumps. The best jump was selected to be later analyzed. Evaluations were done 2 h before the race began and then immediately after the race finished (approximately 1 to 5 min after the race). The protocols of each jump test were applied by following the original protocol proposed by Bosco et al. [39]. 

In order to perform SJ, the participants started in a squatting position with knees bent at 90° and arms on hips to avoid influencing the jump. A goniometer was used to verify the knee angle. The participants had to remain in this squatting position for 3 s before performing SJ. For CMJ, the subjects started from an upright standing position with hands on hips to avoid any arm movement. Then as a single sequence, they made a swift downward movement, followed immediately by a rapid vertical movement to jump as high as possible. Finally, during the ABK test, the participants had to begin by squatting and flexing their knees 90°, followed by swinging their arms to help them to jump as high as possible.

### 2.6. Blood Samples

Twenty milliliters of venous blood (antecubital vein) was withdrawn from each participant in both the pre- and post-race evaluations (90 min before and 10 min after finishing the race). Blood samples were collected in two 5-mL Vacutainer tubes (Vacutainer, beliver industrial state, plymouth PL6 7BP, United Kingdom) without anticoagulant for serum isolation and in two 5-mL tubes containing ethylenediaminetetraacetic acid (EDTA) as an anticoagulant. Once collected, blood samples were coagulated for 25–30 min at room temperature and then centrifuged at 2500 rpm for 10 min to remove the clots. Serum samples were aliquoted into Eppendorf tubes (Eppendorf AG, Hamburg, Germany), previously washed with diluted nitric acid, and conserved at −80℃ until the biochemical analysis. 

### 2.7. Determining Muscle Damage Markers and Hydration Status

A 2-mL blood sample was used to determine leukocytes (leu) with an analyzer model Coulter model AcT diff. The rest, a 3-mL blood sample, was employed to determine creatinine (Cr), alanine aminotransferase (ALT), creatine kinase (CK), sodium (Na) and bilirubin (BIL) by spectrophotometric techniques. Complete biochemistry was processed in the laboratory of the San Jorge University Hospital by a Chemistry Analyzer model Advia 1650 (Bayer, Germany).

### 2.8. Statistical Analysis

Data were processed with IBM SPSS 25.0 Statistics for Macintosh (IBM Corp., Armonk, NY, USA). A descriptive analysis was performed to show the means and standard deviations. The variables’ normality distribution was analyzed by the Shapiro–Wilk test and the homogeneity of variances by the Levene test. The Student’s t-test was applied to determine differences in the participants’ characteristics and the percentages of change (pre-race vs. post-race). A two-way ANOVA (group effect + race effect) was used to show differences between the studied variables. Effect size was calculated for the two-way ANOVA using partial eta-squared (η^2^) as a low effect (0.01–0.06), moderate effect (0.06–0.14) and high effect (>0.14) [42]. The *p* < 0.05 differences were considered statistically significant.

## 3. Results

Table 2 shows the characteristics of the participants in both groups. Significant differences existed in weekly training hours, annual slope achieved while training, times recorded during the GSUR test, maximum speed and percentage of fat (*p* < 0.05).

Table 3 and Table 4 indicate the changes in neuromuscular function, muscle damage and dehydration before and after the race, as well as intergroup differences. For these differences, we observed significant differences for ALT and bilirubin (*p* < 0.05). For the effect that racing had, we found significant differences in SJ, CMJ, ABK, CK, ALT, BIL, leu and Cr (*p* < 0.001). Finally, we observed significant differences in bilirubin when the group x race interaction was analyzed (*p* < 0.05).

**Figure 2:**
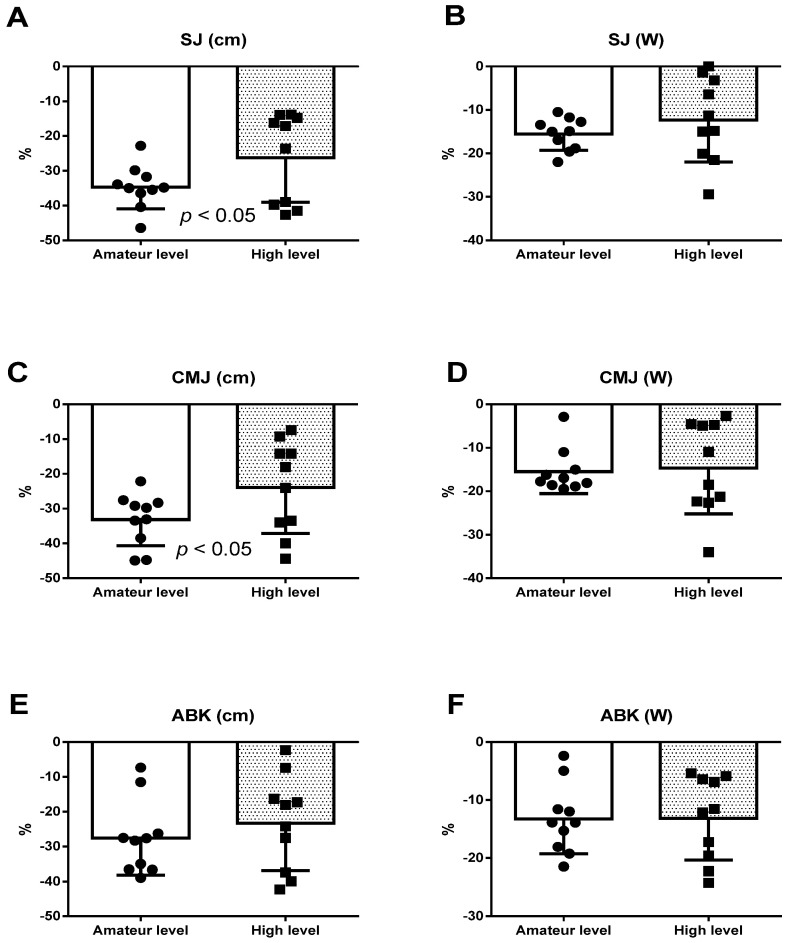
(**A**) Percentage of change in height for SJ; (**B**) percentage of change in power for SJ; (**C**) percentage of change in height for CMJ; (**D**) percentage of change in power for CMJ; (**E**) percentage of change in height for ABK; (**F**) percentage of change in power for ABK; black circle: values of amateur levels participants; black square: values of high levels participants; SJ: squat jump; CMJ: countermovement jump; SJ: squat jump; ABK: Abalakov jump.

**Figure 3:**
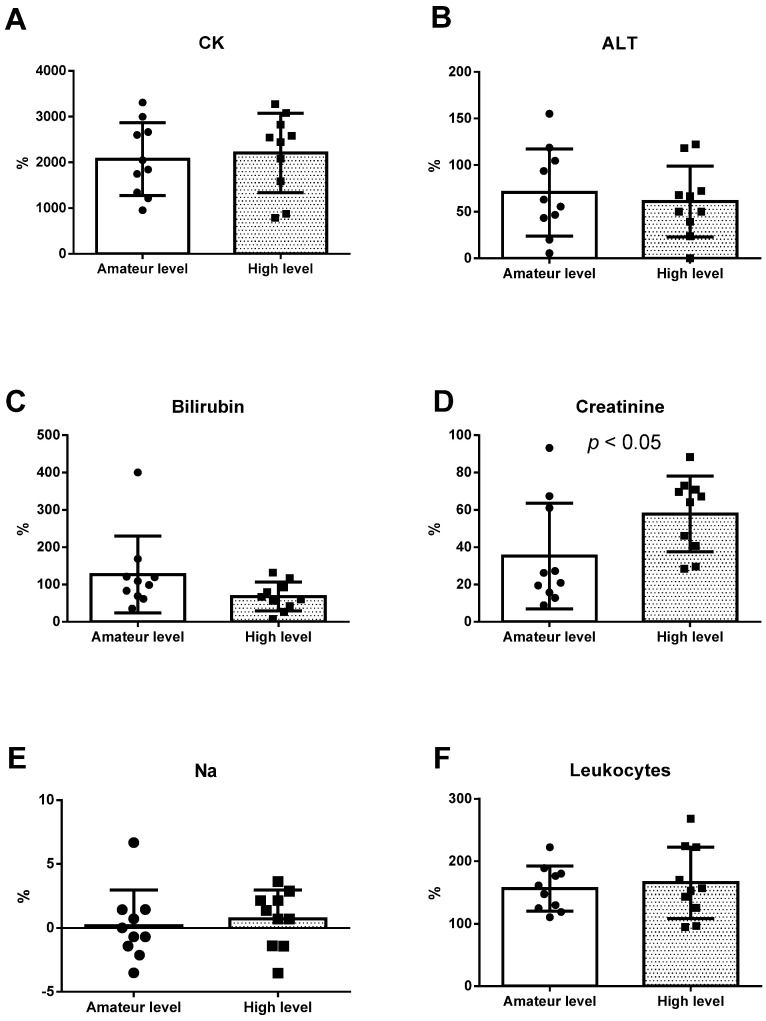
(**A**) Percentage of change in CK; (**B**) percentage of change in ALT; (**C**) percentage of change in bilirubin; (**D**) percentage of change in creatinine; (**E**) percentage of change in Na; (**F**) percentage of change in leukocytes; black circle: values of amateur levels participants; black square: values of high levels participants; CK: creatinine kinase; ALT: alanine aminotransferase; Na: sodium.

Figure 2 and Figure 3 depict the percentages of change (pre vs. post) of the previously studied parameters. Figure 2 illustrates the significant differences in the height of both SJ and CMJ, and loss in the high-level group was lower (*p* < 0.05). Figure 3 shows differences in creatinine (*p* < 0.05), for which a more marked increase was observed in the high-level group.

## 4. Discussion

The present study’s objectives were to: (a) evaluate the acute responses of running an ultratrail race in terms of neuromuscular function, muscle damage and hydration status; (b) analyze if responses could differ according to training level. The GSUR reduced the power and height of the SJ, CMJ and ABK jumps for all the participants. The muscle damage and dehydration markers increased in both groups. However, the percentages of loss in the SJ and CMJ heights were lower in the high-level group than in the amateur-level group (*p* < 0.05). The percentage of increase in creatinine levels was higher in the high-level group (*p* < 0.05). Research concerning the study of trail running has increased in recent years [43,44,45,46]

In general, the responses observed during the GSUR were similar to those reported in other races [12,13,47,48]. Likewise, the study participants presented similar characteristics to runners, as reported by former studies [22,49]. According to similar studies, ultratrail racing could have a distinct impact, particularly for neuromuscular and creatinine parameters, depending on training level [31,32].

For the neuromuscular function parameters, significant reductions in the height and power of all jumps were noted after the race (Table 3). These results fall in line with those previously reported by other authors. For instance, Martínez-Navarro et al. [50] observed lower SJ heights after races covering 65 km and 107 km. Balducci et al. [51] reported a 20% loss in CMJ after a 75 km race, as Gatterer et al. did [52]. However, Rousanoglou et al. [53] observed no significant differences in CMJ height after a mountain marathon race until 5 after min after finishing the race. Apart from ultratrail races being long-distance runs, they involve marked slopes, which implies engaging a high eccentric force component [47] that can cause high levels of muscle damage and fatigue which, in turn, have been associated with excitation–relaxation coupling failure [54,55]. Excitation–contraction coupling failure results in a lower free calcium level in the cytosol, and thus, in less muscle power [55,56]. Hence, ultratrail endurance mountain racing events strongly impact peripheral fatigue by diminishing muscle function and performance [22,56]. The lower jump heights after racing were expected due to functional alterations and the reduced capacity to produce maximum power induced by racing characteristics [13,53]. On this matter, Millet et al. [22], Giandolini et al. [54] and Saugy et al. [21] respectively observed how voluntary maximum knee extensor contraction diminished (35%, 35% and 13%), as did foot flexor contraction (39%, 28% and 10%), after mountain races. In these circumstances, it is not surprising that jump tests are often employed to evaluate neuromuscular fatigue in muscle groups [57,58,59], especially fatigue induced by ultradistance tests. In fact, after repeatedly applying jump tests after a 90-kilometer race, Chambers et al. [60] reported significant drops in SJ and CMJ heights, which remained for 18 consecutive days after the competition. 

Our study also observed how fatigue affected the amateur-level group runners to a greater extent because the reductions in the SJ (Figure 2A) and CMJ (Figure 2C) heights were significantly more marked in this group. These results fall in line with what El-Ashker et al. indicated [61], who found that the subjects with a higher level of training presented better neuromuscular function with fatigue. Nonetheless, these intergroup differences were not noted for either the ABK jump (Figure 2E) or developed power (W) in all jumps (Figure 2B,D,F). These results coincide partly with those from the meta-analysis carried out by Claudino et al. [62] because the achieved CMJ height seemed to better define the degree of the neuromuscular fatigue of knee extensors than other jumps and kinetic parameters, which showed a higher coefficient of variation, such as developed power. 

For muscle and liver damage and inflammation, the present study observed higher CK, ALT and bilirubin concentrations after racing. The obtained results fall in line with those reported for mountain races covering different distances: 43 km [63], 54 km [13], 217 km [48], 280 km [64] and 330 km [65]. The CK and liver enzyme levels, among others, were the most widespread markers to indicate skeletal muscle damage [66]. According to data on jump tests, lower jump heights could be related to high CK and ALT levels in blood [67]. This relation suggests that continuous eccentric actions performed on negative slopes might damage muscle fibers, which leads to the release of muscle proteins in the bloodstream, such as CK [13,68]. The fact that ALT rose could be related to both muscle damage caused by eccentric contractions and liver cell lesions [69]. However, it is known that ALT concentrations are higher in the liver and kidneys, and lower in skeletal muscle [70]. It is also known that the degree of liver lesion is proportional to workload [71]. Likewise, a rise in bilirubin levels after a race has been found by others studies [72,73,74]. Increased bilirubin can be caused by hemolysis produced mainly by mechanical factors and the effect of free radicals [75]. The percentage increase in bilirubin in the amateur level group could have been due to the lower amount of antioxidants in this group compared to the high-level group [75]. As an adaptation, regular physical training increases the levels of antioxidants in the body, increasing the efficiency of the antioxidant system [76,77]. The radicals produced during exercise could increase hemolysis and bilirubin levels.

The rising levels of leukocytes observed in the present study fall in line with previous studies [64,78,79]. Leukocytosis caused by running could be due to increased inflammation caused by muscle damage [80,81]. Acute physical exercise can lead to more cardiac output, vascular vasodilatation and blood flow, which exert stronger mechanical forces on the endothelium which, in turn, leads to leukocytes separating from the endothelium and entering circulation [82]. Moreover, catecholamines and cortisol are secreted during exercise, which could also contribute to increase leukocytes [81,83]. 

No differences were observed in the plasma Na concentration for hydration status. Creatinine levels rose after racing (*p* < 0.001) and the percentage of change was higher for the high-level group (*p* < 0.05). These differences are probably related to strong kidney and muscle impacts. After ultratrail races, runners generally suffer dehydration and body weight loss [1,84]. Drinking plenty of liquids during races is recommended to avoid dehydration [1]. Different hydration strategies are used during ultratrail races to avoid dehydration [85]. The problems of dehydration regarding metabolic and neuromuscular performance were previously outlined [25,28]. Acute kidney lesions are often reported after endurance races, with a prevalence of up to 80% during ultratrail running, but most do not pose serious health problems, and kidney function normally recovers completely after a few days [86,87]. The present study found no significant differences between drinking liquids while racing, unlike some other studies [88]. This could be related to no differences being found in Na concentrations. Plasma Na concentration is the most frequently used parameter to evaluate dehydration status [89]. Former studies reported increased Na after trail races [90,91]. The fact that our study found high creatinine concentrations in runners coincides with previous studies [87,92]. These changes would probably have a multifactorial basis, to which both dehydration and a lower exercise-related glomerular filtration rate would contribute [93]. The fact that serum creatinine concentration positively correlates with lean muscle mass and varies with training type has been demonstrated [88,94].

The study has its limitations: (a) its sample size was small; (b) food intake while racing was not controlled, which could have affected the muscle and neuromuscular damage markers, as other studies have reported [56,95]; (c) only the male gender was studied; (d) it did not evaluate the same markers while the race was underway or in post-race hours to evaluate evolution. As each ultratrail race is unique, these results cannot be completely extrapolated to other races. 

## 5. Conclusions

Ultratrail racing lowers neuromuscular function and increases muscle damage/inflammation. High-level runners suffer less neuromuscular fatigue than amateur-level runners. However, the creatinine levels in the high-level group increased more in metabolic terms.

It is worth knowing the impacts of trail racing in order to schedule and adapt training sessions according to race demands. According to the obtained results, training programs and racing strategies must include muscle damage prevention to reduce ultratrail runners’ muscle fatigue.

## Figures and Tables

**Table 1:** Characteristics of our participants.

Parameters	Amateur Level (*n* = 10)	High Level (*n* = 10)	*p*
Age (years)	43.30 ± 4.52	41.40 ± 6.18	0.443
Height (m)	1.77 ± 0.06	1.76 ± 0.06	0.780
Experience (year)	5.80 ± 2.52	4.60 ± 1.26	0.196
Weekly training (h)	6.50 ± 0.70	11.05 ± 2.94	<0.001
Annual slopes (m)	33,716.7 ± 4427.7	56,426.6 ± 8184.6	0.001

**Table 2:** Body composition, physical tests and result during GSUR.

Parameters	Amateur Level (*n* = 10)	High Level (*n* = 10)	*p*
Time during the race (h)	19.87 ± 1.84	15.31 ± 0.81	<0.001
VO_2max_ (L/min)	4.11 ± 0.37	4.46 ± 0.45	0.079
HR_max_ (bpm)	179.50 ± 7.20	177.29 ± 6.76	0.605
Maximum speed (km/h)	15.00 ± 0.81	16.40 ± 0.96	0.003
Fat mass (%)	10.72 ± 2.28	8.83 ± 1.45	0.040
Muscle mass (%)	43.40 ± 3.65	45.07 ± 2.93	0.276
Water intake (L)	9.64 ± 3.08	11.17 ± 3.79	0.337

VO_2max_: maximal oxygen consumption; HR_max_: maximum heart rate.

**Table 3:** Neuromuscular function before and after the race according to training level.

Parameters	Time	Amateur Level (*n* = 10)	High Level (*n* = 10)	Group Effect	η^2^	Race Effect	η^2^	Group x Race	η^2^
SJ (cm)	Pre	25.50 ± 4.38	27.12 ± 5.29	0.102	0.072	<0.001	0.470	0.597	0.008
	Post	16.74 ± 3.91	19.87 ± 4.31						
CMJ (cm)	Pre	29.86 ± 5.08	31.14 ± 6.33	0.182	0.049	<0.001	0.430	0.554	0.010
	Post	20.11 ± 5.01	23.39 ± 4.64						
ABK (cm)	Pre	34.08 ± 6.44	36.66 ± 8.93	0.189	0.047	<0.001	0.360	0.922	0.000
	Post	24.52 ± 4.88	27.51 ± 5.25						
SJ (W)	Pre	810.80 ± 78.87	821.20 ± 91.68	0.638	0.006	<0.001	0.327	0.906	0.000
	Post	684.80 ± 71.57	702.20 ± 120.62						
CMJ (W)	Pre	876.90 ± 84.09	866.00 ± 107.53	0.659	0.005	<0.001	0.290	0.420	0.018
	Post	740.10 ± 71.11	777.20 ± 103.15						
ABK (W)	Pre	935.30 ± 79.98	950.20 ± 106.14	0.663	0.005	<0.001	0.333	0.949	0.000
	Post	811.80 ± 95.25	822.90 ± 90.97						

CMJ: countermovement jump; SJ: squat jump; ABK: Abalakov jump; η^2^: eta-squared.

**Table 4:** Weight, muscle harm parameters and hydration status before and after the race according to training level.

Parameters	Time	Amateur Level (*n* = 10)	High Level (*n* = 10)	Group Effect	η^2^	Race Effect	η^2^	Group x Race	η^2^
Weight (kg)	Pre	76.01 ± 10.84	75.23 ± 6.87	0.640	0.006	0.474	0.014	0.846	0.001
	Post	74.52 ± 10.21	73.58 ± 8.56						
CK (U/L)	Pre	164.30 ± 69.39	193.60 ± 42.11	0.074	0.086	<0.001	0.817	0.091	0.077
	Post	3251. 60 ± 1011.89	4261.50 ± 1469.60						
ALT (U/L)	Pre	18.60 ± 2.50	23.50 ± 3.92	0.017	0.147	<0.001	0.537	0.904	0.000
	Post	31.70 ± 9.67	37.10 ± 7.46						
BIL (mg/dL)	Pre	0.597 ± 0.148	0.600 ± 0.156	0.028	0.128	<0.001	0.698	0.024	0.133
	Post	1.219 ± 0.205	0.967 ± 0.169						
Leu (×10^3^/ μL)	Pre	5.93 ± 1.00	5.99 ± 0.93	0.589	0.008	<0.001	0.843	0.650	0.006
	Post	15.04 ± 2.29	15.72 ± 3.35						
Cr (mg/dL)	Pre	0.900 ± 0.137	0.855 ± 0.101	0.380	0.021	<0.001	0.634	0.081	0.082
	Post	1.202 ± 0.239	1.335 ± 0.107						
Na (mmol/L)	Pre	140.10 ± 2.33	140.70 ± 1.76	0.194	0.046	0.432	0.017	0.599	0.008
	Post	140.30 ± 2.11	141.70 ± 3.12						

CK: creatinine kinase; ALT: alanine aminotransferase; BIL: bilirubin; Leu: leukocytes; Cr: creatinine; Na: sodium; η^2^: eta-squared.

## Data Availability

Information about the UTGS race is available at http://utgs.es/ (accessed on 11 May 2021).
